# Haptic-Based Perception-Empathy Biofeedback Enhances Postural Motor Learning During High-Cognitive Load Task in Healthy Older Adults

**DOI:** 10.3389/fmed.2018.00149

**Published:** 2018-05-16

**Authors:** Kazuhiro Yasuda, Kenta Saichi, Hiroyasu Iwata

**Affiliations:** ^1^Research Institute for Science and Engineering, Waseda University, Tokyo, Japan; ^2^Graduate School of Creative Science and Engineering, Waseda University, Tokyo, Japan

**Keywords:** older adults, postural control, sensory integration, haptic-based perception-empathy biofeedback, interpersonal feedback, motor learning

## Abstract

Falls and fall-induced injuries are major global public health problems, and sensory input impairment in older adults results in significant limitations in feedback-type postural control. A haptic-based biofeedback (BF) system can be used for augmenting somatosensory input in older adults, and the application of this BF system can increase the objectivity of the feedback and encourage comparison with that provided by a trainer. Nevertheless, an optimal BF system that focuses on interpersonal feedback for balance training in older adults has not been proposed. Thus, we proposed a haptic-based perception-empathy BF system that provides information regarding the older adult's center-of-foot pressure pattern to the trainee and trainer for refining the motor learning effect. The first objective of this study was to examine the effect of this balance training regimen in healthy older adults performing a postural learning task. Second, this study aimed to determine whether BF training required high cognitive load to clarify its practicability in real-life settings. Twenty older adults were assigned to two groups: BF and control groups. Participants in both groups tried balance training in the single-leg stance while performing a cognitive task (i.e., serial subtraction task). Retention was tested 24 h later. Testing comprised balance performance measures (i.e., 95% confidence ellipse area and mean velocity of sway) and dual-task performance (number of responses and correct answers). Measurements of postural control using a force plate revealed that the stability of the single-leg stance was significantly lower in the BF group than in the control group during the balance task. The BF group retained the improvement in the 95% confidence ellipse area 24 h after the retention test. Results of dual-task performance during the balance task were not different between the two groups. These results confirmed the potential benefit of the proposed balance training regimen in designing successful motor learning programs for preventing falls in older adults.

## Introduction

A previous study reported that 30–60% of healthy older adults experience falls annually and that 10–20% of such falls can result in injury, hospitalization, or death (1). Additionally, falls are known to cause loss of independence, a decline in the health status, and a decrease in quality of life (2). Moreover, fall-related injuries have been shown to be associated with high economic cost (3). Recurrent falls and impaired balance are among the most important risk factors for falls and should, thus, be addressed in fall prevention programs. Risk factors for postural instability in older adults vary and encompass various diseases, including abnormalities in balance and gait (4). Woollacott has suggested that borderline pathology in sensorimotor processes plays an important role in postural instability (5). Thus, the interplay between postural and voluntary movements on the basis of sensory control is significant in the performance of motor tasks (6). In this context, sensory input impairment in older adults must result in significant limitations in feedback-type motor control (7). For example, deficits in lower extremity joint proprioception or foot plantar sensory were found to be highly correlated with postural stability (8).

The technique of sensory substitution involves the use of a sensory modality to replace or augment another sensory modality (9). Previous studies have proposed various sensory substitution devices that provide auditory, vibrotactile, and multimodal biofeedback (BF) for countering age- and disease-related imbalance and decreasing fall risk (10–15). Vibrotactile feedback (VTF) was developed to provide individuals having balance problems with external information about their body movements in space. Generally, vibration cues are provided as feedback when body movements exceed a predefined threshold. The effects of VTF applied to the trunk for reducing postural sway in young, healthy subjects, and individuals with vestibular deficits have been validated in several previous studies (16–19). Moreover, studies have shown that VTF can reduce trunk tilt and improve gait performance in older adults (15, 20, 21). Although a series of previous studies have substantiated the efficacy of VTF to a certain extent, recent studies have indicated that VTF can affect postural performance during dual tasks and can affect results of cognitive tasks (20, 22). This is probably because VTF requires participants to engage in higher cognitive processes to deal with the stimulus input. Specifically, older adults would be susceptible to decreased dual-task performance during postural control and gait tasks (23, 24).Thus, when attempting to apply VTF to actual daily life scenarios, this challenge should be seriously considered.

Studies involving skill science have reported that provision of summarized knowledge of results (KR) can help with motor learning (25, 26). Furthermore, previous studies have shown that learners desire to receive positive feedback after a “good” attempt at a task, and the findings indicate the function of feedback as a motivational tool (27–29). The use of BF might improve the quality of summarized KR or encouragement, as this approach allows objective observation of the motion characteristics of a trainee, according to BF information. However, no report has proposed an optimal BF system that would contribute to the refinement of interpersonal feedback between a trainer and trainee. The present study aimed to propose a haptic-based perception-empathy BF system capable of providing data on the center-of-foot pressure (CoP) pattern during training to the trainee and trainer for enhancing the motor learning effect. First, the purpose of the present study was to investigate the impact of the proposed balance training regimen in healthy older adults performing postural learning task. Second, we aimed to reveal the effect of the proposed BF system on the cognitive load during balance training because this aspect is essential for clarifying its feasibility in real-life or clinical setting. We hypothesized that a balance training regimen using the proposed system would enhance balance motor learning in older adults.

## Materials and methods

### Participants

Recruitment of participants was conducted in the Shinjuku-ward, Tokyo area, and facilitated by the Shinjuku Silver Human Resources Center through advertising in local recruitment papers. At the initial visit, a screening physical examination and hearing test were performed. Inclusion criteria were age ≥65, having sufficient communication abilities to understand the instructions provided by the experimenter, living independently in the community, being able to maintain balance in a single-leg stance for >30 s, being able to walk without an aid, being free of neurological or musculoskeletal issues that might influence postural control or cognition (cerebrovascular accident, brain trauma, Parkinson's disease, acute illness, significant orthopedic disability, etc.), having a mini-mental state examination score >20 (no dementia), and having the ability to sense vibrations of the BF system. Moreover, participants were excluded if they had severe visual impairment or hearing deficit, nerve damage, body pain, a history of fainting, or a body mass index >30 kg/m^2^.

Twenty older adults participated in this study, and the participants were randomly allocated to the BF or control groups by a third-party institution (Silver Human Resources Center). An initial sample size of 8 per group was suggested for the pilot study with G-power (30). This was later amended to 10 per group so that the study could be completed with human resources.

### Ethical statements

All procedures were approved by Waseda University Ethics Committee for Human Research. Prior to participation, each participant signed an informed consent form approved by an institutional review board (approval number: 2012-247).

### BF system overview

**Figure 1:**
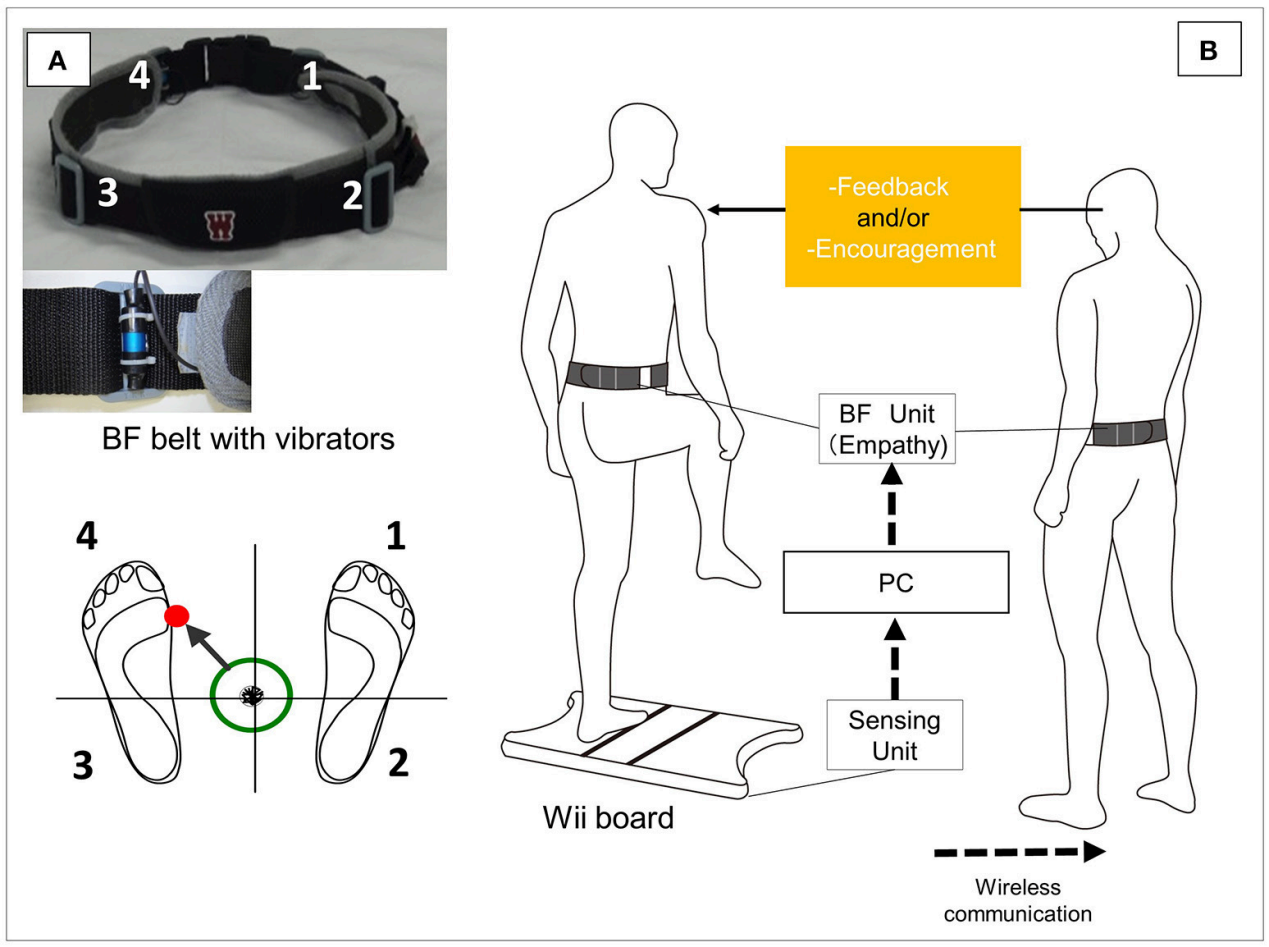
Overview of the biofeedback system. **(A)** When the center-of-foot pressure (CoP) exceeds the predefined threshold area, vibrators on the participant's pelvic belt are activated in the corresponding CoP direction (as indicated in the illustration, when CoP shifts to the front left, the vibrator on the front left is activated). **(B)** During balance training, the vibrators on the trainee's and trainer's pelvic belts are simultaneously activated corresponding to the trainee's CoP direction. Based on the shared information, the trainer is able to provide appropriate feedback and encouragement.

The BF system includes a Nintendo Wii balance board (WBB) (Nintendo Co., Ltd., Kyoto, Japan), a wearable vibrotactile BF device, and a personal computer having a custom-programmed software (Visual Studio; Microsoft Corp., Redmond, WA, USA). The software allows the BF threshold to be recorded and manipulated. We selected the WBB to ensure high usability in a clinical environment, as previous studies have shown that the WBB has good-to-excellent reliability with regard to functional balance performance in healthy older adults (31, 32). The wearable BF device assesses the perception of the pattern of CoP displacement at the pelvis (i.e., anterior and posterior superior iliac spine) during the postural task (Figure 1A). To alleviate cognitive loads on older adults and detect the direction of CoP motion, only four tactile vibrators were used around the participant's pelvis (Figure 1A). Each vibrator (frequency: 80 Hz) was activated when CoP exceeded a predefined circular threshold (33). We used the following approach to determine the predefined circular threshold (33). First, the 95% confidence circle area (34) was measured during a 30-s stance. Second, the target area was defined by 90% of the premeasured 95% confidence circle area. Using this value, the activated vibrators made the participants aware of the direction of their body sway. In older adults, deficits in lower extremity joint proprioception or foot plantar sensory were found to be highly correlated with postural stability (8). In the present BF system, body sway data is augmented with vibrators that are attached to the pelvic girdle.

This system provides BF on the CoP pattern during training to not only the trainee but also the trainer (physical therapist or coach) (Figure 1B). Thus, the postural sway pattern information is shared. Studies have indicated that appropriate instructions to modify movement patterns (35–37) and appropriate motivation for motor learning should be provided to participants (27, 28). However, it is not possible to objectively understand the features of correct movement patterns in real time according to only a trainer's observation. In particular, it is not possible to detect subtle postural movements. Thus, instructions tend to depend on a trainer's experience. As this system shares BF information, it (a) enables the trainer to precisely monitor the trainee's CoP patterns and provide accurate summary feedback and (b) makes it possible to effectively provide encouraging feedback by sharing the information that the balance performance has achieved the objective. Thus, all trainers can accurately and immediately instruct and encourage participants during balance training based on objective BF information, not on subjective evaluation.

### Protocol and postural task

In this study, we selected single-leg stance tasks to observe their effects on postural motor learning because single-leg stance tasks are more challenging tasks that are predictive of balance problems and better indicators of fall risk in older adults (38–40).

**Figure 2:**
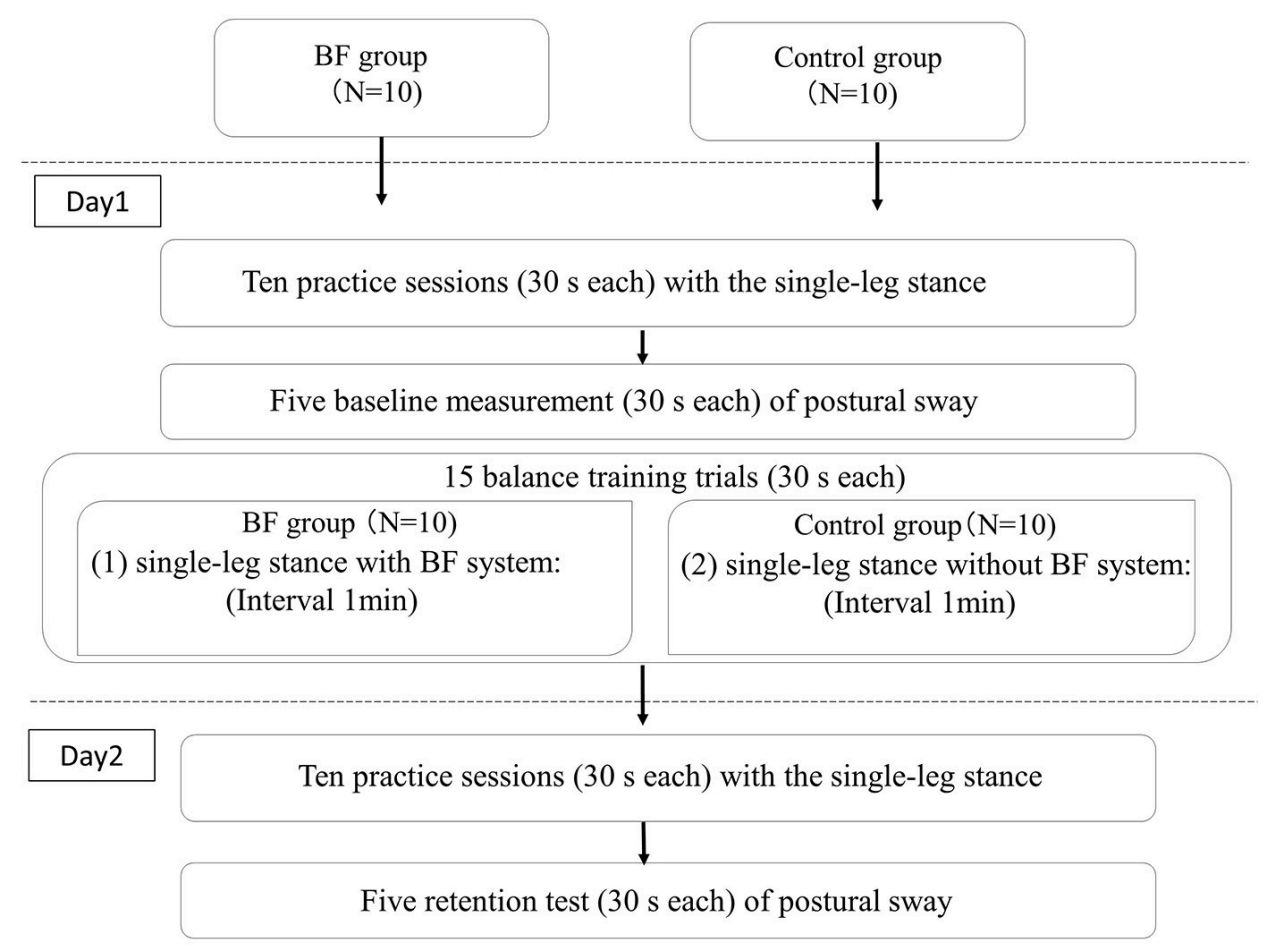
Flow chart of the experimental procedure.

Measurement of postural sway comprised three phases: baseline test, postural training (day 1), and retention test (day 2) (Figure 2). Before the baseline test evaluation, participants were given 10 practice sessions (30 s each) for familiarization with the single-leg stance task. After this practice session, five baseline measurements (30 s each) of postural sway in the single-leg stance were obtained as a baseline test. The predefined circular threshold was determined at the last baseline measurement.

fixed eye-level target test, participants proceed to the postural training session. Each participant stood barefoot on the WBB with eyes open while looking at a fixed eye-level target placed ~2 m away. A training session is composed of 15 performances (30 s each), with a rest interval of 1 min between each performance. For both groups, the participants were asked to minimize postural sway to avoid activating the vibrators.

To synchronize the lengths of time required for providing feedback to the BF and control groups, feedback was only provided once every three trials during 1-min intervals (5 min in total). The BF group received specific feedback from the trainer, and encouragement was provided during training sessions. Shared haptic feedback information was only used to provide feedback and encouragement. Specifically, feedback regarding the body sway pattern was provided (e.g., “The body is leaning toward the forward right during training,” “It takes time to return to the left side from the right side,” or “The body is swaying forward and backward on the right side”). If appropriate balance was achieved, encouragement was provided with words such as “good” and “that's better.” The control group also underwent postural control tasks under the same experimental conditions, except the use of BF. The control group received feedback about postural sway pattern every three trials. This feedback and encouragement were based on the observation of the trainer. Feedback to the participants was provided by the same licensed physical therapist.

On day 2 (~24 h between visits), the participants underwent the same initial approach (10 postural tasks). Then, a retention test was performed. It involved five tasks (30 s each), with a rest interval of 1 min between tasks.

### Secondary cognitive task: a digit subtraction task

To evaluate the effect of dual tasks during the postural tasks, a digit subtraction task was used during the single-leg stance task (41, 42). The participants were told to count aloud backward by seven from a number that was determined through the selection of a card with a random number from 125 to 250. For each postural trial, the participants were told to randomly select a different card from a pack of cards. The participants were shown the selected card for 5 s before the start of the balance task. There were no practice or familiarization trials for the additional task in order to prevent the participants from learning the secondary task.

### Outcome measurements

#### Postural performance

Two representative dependent variables were used for describing the participant's postural stability (43). First, 95% confidence ellipse area was used as a measure of CoP spatial variability (43). It represents the 95% bivariate confidence ellipse area that is expected to cover about 95% of points on the CoP path. Second, the mean velocity of CoP displacement (mm/s) was considered. It represents the total distance covered by CoP (total sway path) divided by the test duration (43, 44).

#### Secondary cognitive task

Cognitive performance is considered as the number of correct arithmetic calculations (45). When participants simultaneously performed the cognitive and postural tasks (dual-task trials), dual-task scores were calculated. When participants performed the cognitive task, single-task scores were calculated. During the single task, each participant was told to subtract seven from a random number as many times as possible in 30 s. Verbalizations were recorded from a digital video camera with a built-in microphone and then analyzed in Adobe Premiere Pro CC (Adobe Systems, San Jose, CA).

### Analysis

#### Postural performance

Prior to the analysis, the motor performance score was separately normalized (%) to the baseline for each subject (46). Then, the 95% confidence ellipse area and MV values were normalized to the baseline test for each group. As a result of this normalization, all baseline values for the 95% confidence ellipse area and MV were ≒ 1.

To assess an improvement associated with adaptation on day 1 of training, the postural training session involved 15 trials of the single-leg stance task. Similarly, retention (day 2) was assessed in five trials of the single-leg stance task. Normality was assessed using the Shapiro–Wilk test, and if violation of normality was noted, a non-parametric test was considered. Scores were analyzed in a univariate analysis of variance (ANOVA) (47). For the day 1 training session, scores were analyzed in 2 groups × 15-trial ANOVA with repeated measures on the last factor (47). The retention of improvement was assessed by comparing the results of trials 1–5 on day 1 and the results of the same trials on day 2, using a paired samples *t*-test or the Wilcoxon signed-rank test (48, 49). Alpha was set at *p* = 0.05.

#### Cognitive performance

Prior to the analysis, the number of correct digits verbalized during dual-task trials was normalized to that verbalized during a proportional time period in a single task (i.e., subtraction task in the seating position) (45). A two-tailed, independent *t*-test or Mann–Whitney test was used for determining if cognitive performance in the BF and control groups differed during 15 trials.

## Results

### Participant characteristics

Participants' characteristics are described in Table 1. There were no differences between the BF and control group regarding basic characteristics of the participants.

**Table 1:** Participant characteristics.

	**BF group (*n* = 10)**	**Control group (*n* = 10)**	***p-*value**
Sex (n, females)	5	5	
Age (y)	71.2 ± 2.4	72.7 ± 3.4	0.9370
Weight (kg)	59.0 ± 13.7	55.6 ± 5.0	0.4717
Height (cm)	161.9 ± 6.6	158.5 ± 6.2	0.2472
Leg length (cm)	81.2 ± 4.5	79.3 ± 4.4	0.3729

*Values are denoted in mean ± SD. BF, biofeedback*.

### Postural performance

Results of the two-way ANOVA for the postural sway parameters are depicted in Table 2.

**Table 2:** Two-way ANOVA results of the 95% confidence ellipse area and mean velocity of sway in the experiment.

**POSTURAL SWAY PARAMETERS**
95% CONFIDENCE ELLIPSE AREA (*n* = 10)				
**Source of variation**	**DF**	**MS**	***F***	***P***
Group (A)	1	20.63	53.30	<0.0001^**^
Trials (B)	14	0.1374	2.425	0.0033^**^
A × B	14	0.1888	3.33	<0.0001^**^
Residual	252	0.05665		
Total	281			
MEAN VELOCITY OF SWAY (*n* = 10)				
**Source of variation**	**DF**	**MS**	***F***	***P***
Group (A)	1	1.497	8.80	0.0083^**^
Trials (B)	14	0.07325	9.33	<0.0001^**^
A × B	14	0.02353	2.99	0.0003^**^
Residual	252	0.007855		
Total	281			

*p-value derived from ANOVA for the main effects of group and trials and interaction between group and trials. DF, degrees of freedom; MS, mean square*.

** *p < 0.01*.

#### 95% confidence ellipse area

**Figure 3:**
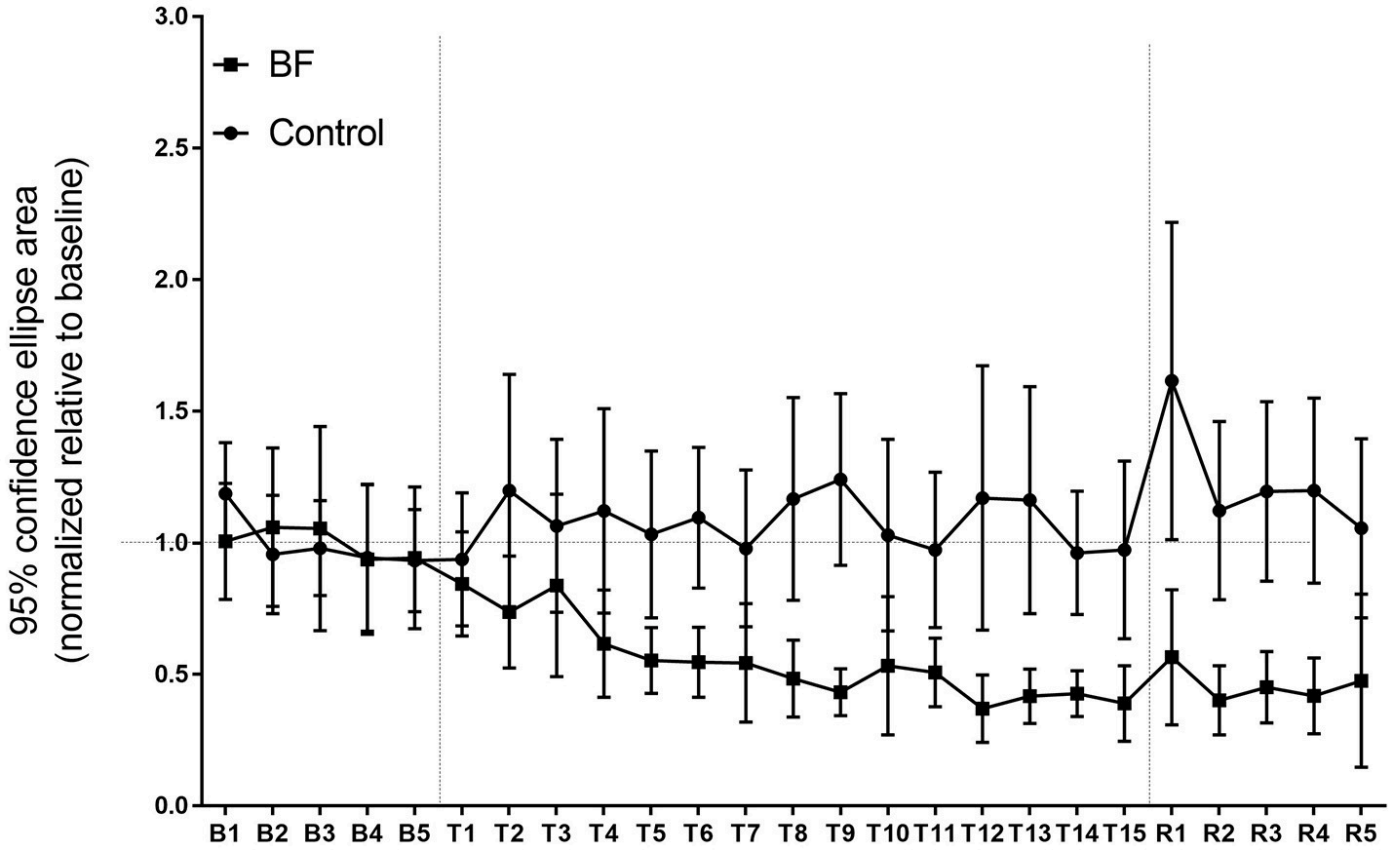
Ninety-five percent confidence ellipse area in the experiment (higher scores reflect greater spatial variability). Error bars indicate standard deviation.

Baseline scores between BF and control groups were not different (Figure 3, left). For the postural performance on day 1, the main effect in the groups was significant [*F*_(1, 18)_ = 53.30, *p* < 0.0001]. Throughout the training, the BF group outperformed the control group (Figure 3, middle). The main effect of the trials [*F*_(14, 252)_ = 2.425, *p* = 0.0033] and interactions between the groups and trials were significant [*F*_(14, 252)_ = 3.333, *p* < 0.0001]. Follow-up analyses indicated that the BF group decreased their sway area from trial 1 to trial 9 (*p* = 0.000138.), from trial 1 to trial 12 (*p* < 0.0001), from trial 1 to trial 13 (*p* < 0.0001), from trial 1 to trial 14 (*p* = 0.000114), and from trial 1 to trial 15 (*p* < 0.0001), whereas the control group showed no change across trials. Adaptations were retained on day 2 for the BF group (*t* = 4.41, *p* = 0.0017) but not for the control group (*t* = 1.48, *p* = 0.1732).

#### Mean velocity of sway

**Figure 4:**
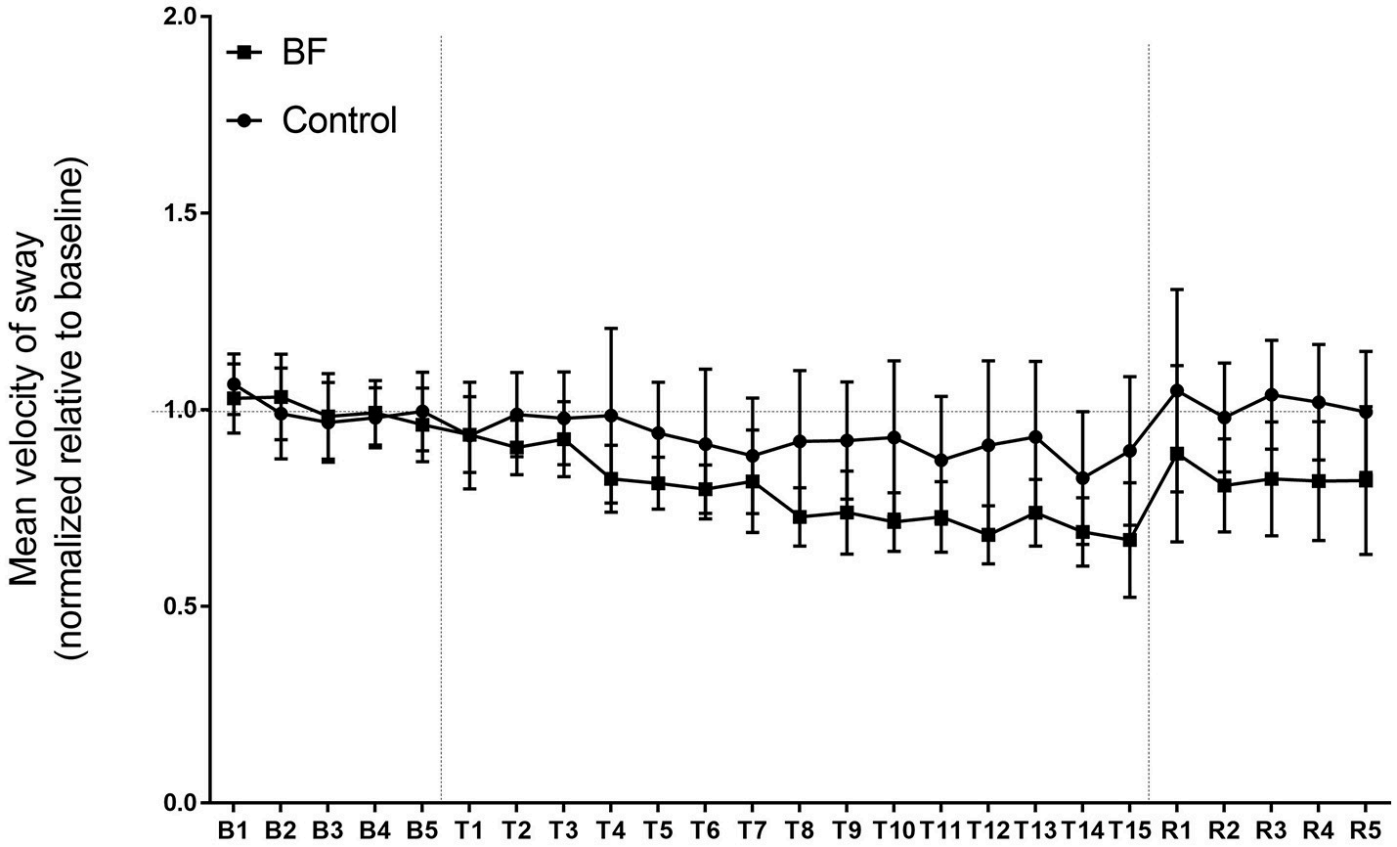
The mean velocity of sway in the experiment (higher scores reflect greater quantity of postural sway). Error bars indicate standard deviation.

Baseline scores were not different between the BF and control groups (Figure 4, left). For postural performance on day 1, the main effect in the groups was significant [*F*_(1, 18)_ = 8.802, *p* = 0.0083]. Throughout the training, the BF group outperformed the control group (Figure 4, middle). The main effect of trials [*F*_(14, 252)_ = 9.325, *p* < 0.0001] and the interaction between the groups and trials were significant [*F*_(14, 252)_ = 2.996, *p* = 0.0003]. Follow-up analyses indicated that the BF group had decreased sway velocity from trial 1 to trial 8 (*p* < 0.0001), from trial 1 to trial 9 (*p* < 0.0001), from trial 1 to trial 10 (*p* < 0.0001), from trial 1 to trial 11 (*p* < 0.0001), from trial 1 to trial 12 (*p* < 0.0001), from trial 1 to trial 13 (*p* < 0.0001), from trial 1 to trial 14 (*p* < 0.0001), and from trial 1 to trial 15 (*p* < 0.0001), whereas the control group had decreased sway velocity from trial 2 to trial 14 (*p* < 0.0001), from trial 3 to trial 14 (*p* = 0.000162), and from trial 4 to trial 14 (*p* < 0.0001). The adaptations were not retained on day 2 for the BF group (*t* = 1.36, *p* = 0.20) and control group (*t* = 1.06, *p* = 0.3426).

### Cognitive performance

**Figure 5:**
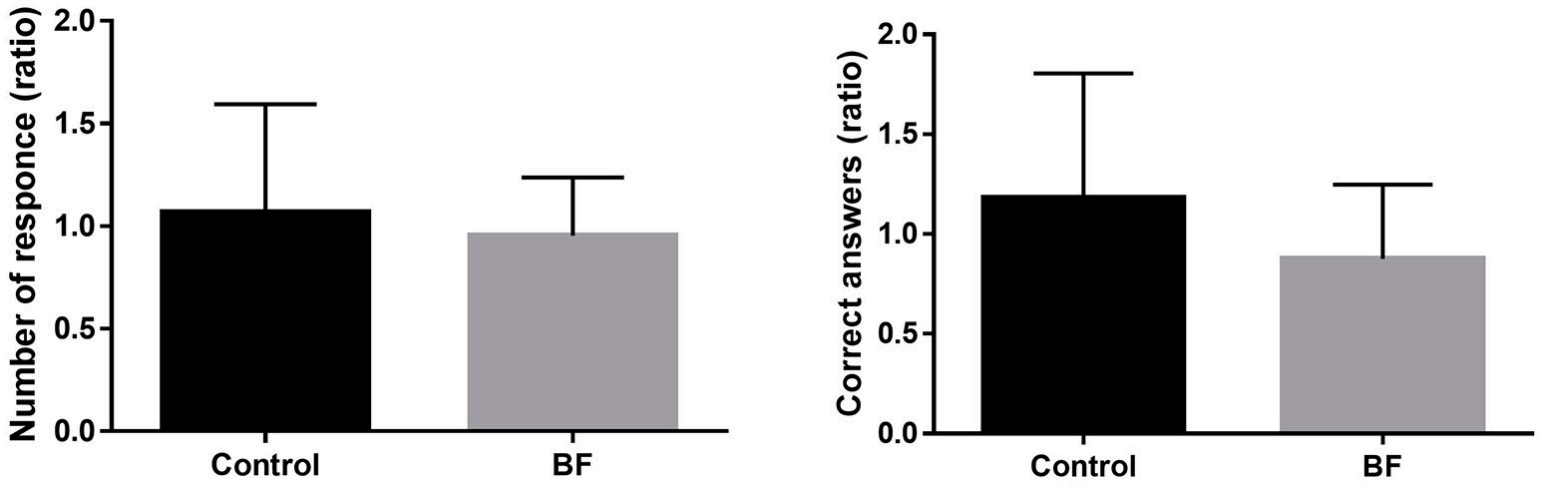
Dual-task costs (as a ratio in performance compared with single-task performance) (mean ± standard deviation).

Regarding cognitive performance, no significant difference was found between the BF and control groups in the number of answer (*t* = 0.60, *p* = 0.5567) and percentage of correct answer (*t* = 1.32, *p* = 0.2004; Figure 5).

## Discussion

This initial study has shown that using the proposed system, the BF group was more effective in adapting to the postural control tasks than the control group, and the older participants retained these improvements over 24 h only for spatial variability, but not for the mean velocity of sway. Moreover, cognitive loads applied to the participants were not significantly different between the BF and control groups, suggesting that the cognitive burden of using the BF system is low. These results increase expectations that the BF system proposed in this study will contribute to developing efficient motor learning modalities incorporating postural control tasks for older people. Indeed, this system is anticipated to be highly practical given its low cognitive burden.

Several factors might have been associated with the beneficial effect with regard to the balance performance observed in this study. The addition of BF might have resulted in an increase in the accuracy of motion correction during BF training (33). Additionally, empathy feedback might have had several effects, including (a) realization of accurate summary feedback from the trainer and (b) strengthening of the reward with encouragement provided by the trainer. With regard to the realization of accurate summary feedback from the trainer, it has been shown that motor learning is facilitated by providing summarized KR (25, 26). The quality of summarized KR might have been improved by empathy BF, as the trainer could objectively determine the motion characteristics of older adults considering the vibration information with empathy BF. With regard to strengthening of the reward with encouragement provided by the trainer, studies involving skill science have shown that learners desire to receive feedback after a good rather than poor attempt, and the findings indicate the function of feedback as a motivational tool (27, 28). Furthermore, rewards have been shown to enhance memory retention across multiple motor learning models in healthy subjects (29). Considering that encouragement can be provided according to the shared BF of an objective task and that encouragement can be provided when the defined exercise target is achieved, shared BF might have enhanced the effect of encouragement as a reward.

In this study, participants solve some mental arithmetic tasks of subtraction during the postural control tasks, and performance of the BF group was compared with that of the control group. Consequently, the use of BF in the BF group did not negatively affect the performance of dual tasks compared with that of the control group. Previous studies found that secondary cognitive tasks (reaction time) significantly increased while using VTF in younger and older adults (20, 22). Older adults had a larger increase in reaction time than younger adults, suggesting that greater attentional demands were required in older adults when using VTF information. Given these results, the authors have pointed out that future training protocols for VTF should consider the effect of aging (20). Our system uses a minimum number of vibration cues (four points) and allows the instructor to utilize and provide BF information identical to the summarized KR. These attributes might have helped mitigate cognitive burden during training. Results of this study apparently suggest that the proposed BF training system can provide training regimens with relatively low cognitive burdens (or negative effects associated with postural control tasks) even for older participants who are susceptible to adverse effects of dual tasks. However, further validation using different sets of tasks is necessary because repercussions of cognitive loads are contingent on the characteristics of each cognitive task (23, 24).

Several limitations have been noted. First, it is impossible to strictly quantify the extent of vibration feedback, and the guidance/feedback derived from BF empathy affected the results of the present study. To clarify this point, basic comparative research on single BF vs. dual BF will be required. Second, the sample size was small, and further rigorous studies with a large sample size are required to confirm these results. Finally, although this study evaluated the feasibility of the device, practice was performed for only 1 day and retention was evaluated for only 24 h. Future studies with a more advanced design are required to assess long-term balance training programs and to investigate whether improvements during training can be retained over a long period.

In conclusion, the present study demonstrated that the BF group more efficiently adapted to the balancing tasks than the control group that did not use BF, and older participants retained the improvement of postural spatial variability over 24 h only for spatial variability but not for the mean velocity of sway. With regard to the cognitive costs during the learning tasks, no significant difference was observed between the BF and control groups, thus suggesting the low cognitive cost of our system. The results indicated that the initial feasibility of the proposed balance training method in enhancing the motor learning effect highlights the potential use of the proposed BF systems in the field or clinical setting.

## Author contributions

KY designed this study, acquired and analyzed data, and drafted the manuscript. KS made substantial contributions to the acquisition and analysis of the data. HI was involved in the conception of the system and design of the study. The authors have read and concurred with the content of the final manuscript. No one who qualifies for authorship has been omitted from the list.

### Conflict of interest statement

The authors declare that the research was conducted in the absence of any commercial or financial relationships that could be construed as a potential conflict of interest.
